# Digital Rehabilitation Following Ultrasound-Guided Injection for Chronic Rotator Cuff Injury: Randomized Controlled Trial

**DOI:** 10.2196/79494

**Published:** 2026-06-10

**Authors:** Zifu He, Yuemin Zhang, Liyan Shi, Gaineng Yuan, Xuefeng Liu, Yue Cai, Jue Zhao, Jing Xu

**Affiliations:** 1 School of Gongli Hospital Medical Technology, University of Shanghai for Science and Technology, Shanghai, China; 2 Pudong Gongli Hospital, Shanghai University of Medicine & Health Sciences, No. 219, Miaopu Road, Shanghai, China, 86 13817512743

**Keywords:** rotator cuff injury, digital therapeutics, rehabilitation exercise, injection therapy, system proficiency

## Abstract

**Background:**

Chronic rotator cuff injuries (CRCIs) often lead to shoulder pain and dysfunction. Although ultrasound-guided injections provide rapid pain relief, subsequent rehabilitation efficacy is often compromised by poor exercise adherence and inaccurate movement execution. Digital therapeutics may enhance patient engagement and movement accuracy, potentially improving outcomes.

**Objective:**

This study compared the effects of ultrasound-guided corticosteroid plus local anesthetic injection combined with the Digital Rehabilitation System training versus traditional exercise on pain intensity and shoulder function in patients with CRCIs.

**Methods:**

This was a single-center, parallel-group, superiority randomized controlled trial with a blinded outcome assessor, injection physician, and data analyst. Sixty eligible participants were randomized to either the conventional treatment group (group C, n=30) or the digital treatment group (group D, n=30) for a 3-month intervention. Following pain-alleviating ultrasound-guided injections, group C performed prescribed exercises, while group D used the intelligent system for self-corrective adaptive training. Primary outcome was the Constant-Murley Score. Secondary outcomes included the University of California Los Angeles Shoulder Scale, Numerical Rating Scale for pain, range of motion (ROM), and system proficiency scores. Assessments occurred at baseline (T1), 1-week (T2), 1-month (T3), and 3-month (T4) follow-ups.

**Results:**

No significant between-group differences existed in baseline characteristics or outcome measures (all *P*>.05). No group × time interactions were observed for any outcomes. At T4, group D demonstrated significantly greater improvements in Constant-Murley Score (Hodges-Lehmann estimator [HLE] −6, 95% CI −10 to −2; *P*=.007), University of California Los Angeles Shoulder Scale score (HLE −2, 95% CI −4 to −1; *P*=.001), and Numerical Rating Scale score (HLE 1, 95% CI 0-2; *P*=.002), and their improvement values (Δ) all exceeded the corresponding minimal clinically important differences. ROM also showed significant intergroup differences in all planes. Compared with T1, ROM was significantly improved in both groups at T4, with the greatest improvement in abduction (Δ group C=20.7 and Δ group D=30.5). Subgroup analysis revealed better enhanced shoulder function and ROM in participants with no or mild shoulder motion restrictions using digital therapy.

**Conclusions:**

For participants with CRCIs, combining ultrasound-guided injection with subsequent Digital Rehabilitation System training achieves greater shoulder ROM and functional scores than traditional exercise after pain relief. This approach demonstrates enhanced efficacy in subgroups with no or mild shoulder mobility limitations.

## Introduction

Chronic rotator cuff injury (CRCI) is the primary cause of shoulder pain and dysfunction, and its prevalence is increasing worldwide in the occupational and geriatric populations [1]. When rotator cuff tendon lesions occur, the shoulder’s stability is disrupted, often leading to persistent pain, limited range of motion (ROM), and reduced muscular strength [2]. If untreated, CRCI can also lead to shoulder stiffness and muscle atrophy, which further exacerbate shoulder dysfunction. Although ultrasound-guided injection therapy has been shown to facilitate tissue repair by providing targeted relief of acute pain, the medium- and long-term improvements in shoulder function are still not promising [3-5]. As a core intervention in the recovery from CRCI [6,7], the effectiveness of exercise is significantly correlated with movement accuracy and compliance [8]. Studies have shown that systematic rehabilitation not only improves shoulder mobility and muscle coordination but also demonstrates a more sustained analgesic effect than local injection therapy, reducing the risk of disability [7,9]. However, based on paper-based guidelines and follow-ups, conventional rehabilitation often leads to secondary injuries or delayed recovery due to adherence to incorrect exercise patterns.

In recent years, the rapid development of digital therapeutics (DTx) has provided innovative solutions in exercise rehabilitation [10-12]. Motion capture technology allows real-time acquisition of participants’ joint kinematic parameters through multisensor fusion with deep learning algorithms. Recently, a study demonstrated that digital interventions using inertial motion trackers integrated with mobile apps exhibit comparable efficacy with traditional in-person supervised programs for patients with shoulder pain, with DTx users showing more substantial clinical improvements in shoulder function [13]. Emerging evidence further validates the effectiveness of digital protocols as novel therapeutic modalities, demonstrating superior efficacy in alleviating pain and associated disability [6,11,14].

However, most existing studies focus on postoperative rehabilitation or acute injury. There is still a gap in the standardization of movement performance for participants with CRCI [15]. This study aims to develop disease-adaptive intelligent assessment models to achieve motion modification based on participants’ movement data and to evaluate the clinical value of ultrasound-guided injection therapy combined with digital therapy for CRCI. The authors hypothesized that this digital protocol after injection therapy would result in a more significant improvement in pain as well as function in participants with CRCI than conventional methods.

## Methods

### Study Design

This study was conducted in accordance with the CONSORT-EHEALTH (Consolidated Standards of Reporting Trials of Electronic and Mobile Health Applications and Online Telehealth) guidelines (Checklist 1) [16]. This prospective, single-center, randomized controlled trial used a 2-arm parallel design. Eligible participants were randomly assigned to either the conventional treatment group (group C) or the digital treatment group (group D) for a 3-month intervention period (March 1 to June 17, 2025). The blinding status (masking of the injection physicians, the outcome assessor, and the data analyst) was maintained until trial completion. Only the investigator generating the randomization sequence and administering the Digital Rehabilitation System was unblinded to group allocation.

### Sample Size Calculation

Prior to recruitment, the minimum sample size was calculated by G*Power 3.1.9.7 to ensure the rigor of statistical analysis [17]. Based on previous studies, the Constant-Murley Score (CMS) was used as the primary outcome in this study. The sample size was calculated based on the between-group difference in the improvement values (Δ) from baseline to postintervention. The minimum difference value between the 2 groups was 5.1 (SD 5.2), resulting in an effect size (*d*) of 0.98 [18]. By using a 2-tailed *t* test (taking α=.05, test efficacy=0.90, an allocation ratio of 1:1, and a 20% dropout rate), a minimum of 58 individuals needed to be included.

### Participants

Participants were recruited at the outpatient pain clinic of Pudong Gongli Hospital, Shanghai University of Medicine & Health Sciences. Both groups were required to receive injections and to undergo rehabilitation after therapeutic patient education. Group C underwent home rehabilitation, while group D underwent rehabilitation through the Digital Rehabilitation System along with home exercises.

Eligible participants comprised adults aged 18 years or older with intact cognitive function and athletic ability who met the diagnostic criteria for rotator cuff injury (RCI) [19,20]. Inclusion required unilateral symptomatic presentation with pain intensity ≥3 on the Numerical Rating Scale (NRS) persisting for at least 3 months, along with a Neer classification of stage II or less [21] diagnosed by magnetic resonance imaging. Exclusion criteria comprised full-thickness tears, active infections, neoplastic conditions, concomitant pathologies affecting shoulder function (eg, shoulder fracture, glenoid labral injury, osteoarthritis, etc), prior ipsilateral shoulder surgery, hypersensitivity to interventional medications, pregnancy or lactation, and individuals with severe audiovisual or motor impairment.

Additionally, participants were stratified into subgroups based on the baseline active range of motion (AROM) of shoulder flexion and abduction, given their primary importance for upper limb function. The classification used clinically meaningful thresholds informed by functional requirements. Individuals with an AROM of ≥150° for both flexion and abduction movements were classified as having no or mild functional limitation (subgroup 1), as this range is typically sufficient for essential overhead activities [22]. Severe limitation (subgroup 2) was defined as an AROM of ≤90° in either flexion or abduction, a threshold associated with substantial disability in performing basic activities of daily living. Participants who did not meet the criteria for subgroup 1 or 2 were classified as having moderate limitation (subgroup 3).

### Allocation and Blinding

Researcher 1 (JX) generated and recorded 60 numbers using the RANDBETWEEN function before the start of the trial and used these numbers to determine a concealed allocation sequence (based on the size of the numbers). This sequence was prepared in a set of sequentially numbered, opaque, sealed envelopes by researcher 1. Researcher 2 (ZJ) was responsible for receiving participants, determining their eligibility, and administering medications with musculoskeletal ultrasonography. After researcher 2 confirmed a participant’s eligibility, researcher 1 opened the next consecutively numbered envelope in the presence of the participant to reveal and execute the allocation. To ensure objectivity and eliminate subjective influence, all outpatient training was monitored by researcher 1, who remained passive and did not intervene in the exercise process. The other outcome indicators were evaluated and recorded by researcher 3 (ZH). The anonymized data were processed and analyzed by researcher 4 (YC). To ensure the objectivity of the results, all investigators outside of researcher 1 were blinded until the end of the study.

### System Equipment

**Figure 1.**
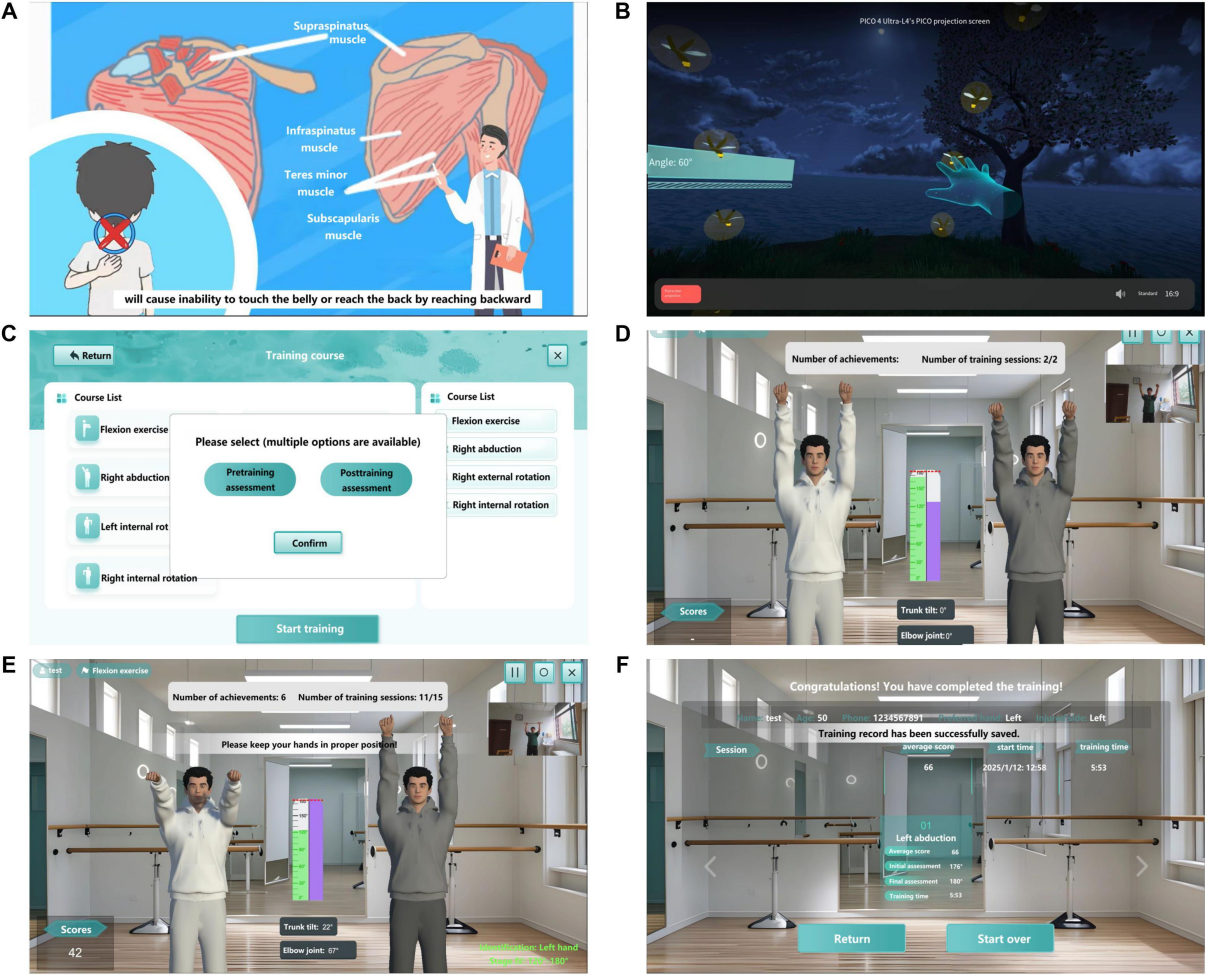
Operating Interface of the Digital Rehabilitation System: (A) therapeutic patient education, (B) butterfly capture task, (C) exercise prescription selection, (D) pretraining assessment for grading, (E) digital training, and (F) training results.

The system was developed by Pudong Gongli Hospital and Ruiku Medical Technology Co, Ltd. It is divided into 2 subsystems: the Virtual Reality (VR) Education subsystem (VRS) (Figure 1A and B) and the Motion Capture subsystem (MCS) (Figure 1C-F). All participants used the VRS for therapeutic patient education and a butterfly capture task, which involved video tutorials about the function of the rotator cuff, the symptoms and causes of RCI, and the methods of exercises [23]. And they needed to use the MCS for training. The background in MCS includes 3 partitions of a virtual coach, an avatar, and a training scene, which serve to guide the exercise, provide real-time feedback, and avoid danger, respectively.

The VRS module was built around a PICO 4 Ultra mixed-reality head-mounted display (PICO, ByteDance Ltd). This device is powered by a Qualcomm Snapdragon XR2 Gen 2 processor and is equipped with a comprehensive sensor array, including dual 32-megapixel RGB cameras and an iToF depth-sensing camera for high-fidelity environmental tracking and stereoscopic visual rendering. The headset provided participants with synchronized audiovisual stimuli. For researcher monitoring and intervention, a Xiaomi Pad 5 tablet (Xiaomi Inc), featuring a Qualcomm Snapdragon 860 platform, was used to run dedicated supervisory software, allowing real-time observation of the participant’s first-person perspective.

The MCS module consisted of 2 primary components: an Azure Kinect Developer Kit (DK) camera (Microsoft Corp) and a Lenovo Legion Y7000 IRX9 laptop computer (Lenovo Group Ltd). The Azure Kinect DK, which features a 12-megapixel RGB camera and a 1-megapixel depth camera, served as the primary sensor for capturing high-precision shoulder joint kinematic data. The laptop, running the Windows 11 operating system and equipped with an Intel Core i7-13650HX processor and NVIDIA GeForce RTX 4060 GPU, hosted the custom analysis software built on the Unity platform. This system compared the captured motion data against a predefined standard movement model in real time and generated immediate graphical and auditory feedback to guide participants in correcting aberrant movement patterns.

### Intervention

#### Overview

All participants received injections administered by the same physician team. The injectate was prepared by first combining 0.25 mL of compound betamethasone injection (containing 1.25 mg of betamethasone sodium phosphate and 0.5 mg of betamethasone dipropionate) with 2.5 mL of 2% lidocaine to form a base solution [3,24]. This base solution was then diluted with 0.9% saline to a total volume of 10 mL. Subsequently, 1 mL of the mixture was discarded, and the remaining 9 mL was used for injection, resulting in a final lidocaine concentration of 0.5%. Based on the magnetic resonance imaging diagnosis, pathological tissues (including structural lesions and inflammatory regions) were precisely targeted under musculoskeletal ultrasound guidance. Participants were positioned supine for subcoracoid bursa and intertubercular groove injections and then placed in a contralateral decubitus position for subacromial bursa injection [3,25,26], administering 3 mL per site. Postprocedural care included sterile dressing application at needle entry points, with instructions to maintain site dryness and avoid strenuous activity for 24 hours. All individuals received 3 injections at 1-week intervals to minimize the potential for corticosteroid-related tendinopathy [27].

Flexion, abduction, external rotation, and internal rotation exercises were prescribed for all participants, with each session lasting 10-15 minutes and administered 3 sessions per day [28]. In addition, all participants attended a weekly follow-up appointment at the outpatient clinic. These sessions were conducted by the same designated physician and physiotherapist to ensure consistency in clinical assessment. Additionally, researcher 1 administered a standardized, interactive session using the VRS to enhance participants’ understanding of their condition and the rehabilitation principles. Separately, participants also completed one of their 3 daily training sessions under supervision at the outpatient clinic on weekdays. The remaining 2 daily sessions were conducted independently at home, performing conventional exercises without the digital system. Participants in group C exercised within the range of pain tolerance.

#### Flexion Training

Participants held a booster stick in both hands and made the shoulder joint flex forward. When the affected shoulder could not continue to flex forward due to weakness or adhesion, the unaffected one assisted or passively lifted it up to its maximal AROM and slowly lowered it down after 15‐30 seconds.

#### Abduction Training

The method was in accordance with flexion training. At the end of AROM, the unaffected arm forcefully retracted to provide assistance, prompting the affected shoulder to the maximum angle within the tolerable range of pain.

#### External and Internal Rotation Training

The unaffected hand applied downward pressure on the head of the affected humerus, and participants were asked to exercise while the affected shoulder was kept in 90° abduction with the elbow flexed to 90°.

Participants allocated to group D used the custom-developed rehabilitation system for their training regimen. Prior to each session, participants performed 2 maximal ROM trials in specified directions to determine their appropriate training phase. Subsequently, the system automatically assigned them to a corresponding phase for 1 exercise set. The exercise modalities within the system were the same as the movements performed by group C. Real-time visual feedback, displayed as a discrepancy between an on-screen coach avatar and the participant’s own motion capture, along with a performance score, allowed for immediate self-correction to enhance movement accuracy [29]. The protocol incorporated an automatic progression rule. This rule stipulated that the system would advance a participant to the subsequent phase once their joint angle consistently met the threshold for the next level [30]. Each set comprised 15 movement repetitions. Following set completion, participants underwent 2 reassessment trials. The total time for 1 system-guided set was 15 minutes. Participants attended 1 in-clinic session on weekdays, supplemented by home-based exercises, for a total of 3 sets daily.

### Outcome Measures

NRS, CMS, and University of California Los Angeles (UCLA) Shoulder Scale scores, and ROMs of forward flexion, abduction, and external and internal rotation of the affected shoulder were collected from each one by the same personnel at baseline (T1), 1-week follow-up (T2), 1-month follow-up (T3), and 3-month follow-up (T4) [31]. In addition, there was a need to score the rehabilitation movements in both groups using the Digital Rehabilitation System at T4. Upon reasonable request, the data supporting the following findings of this study can be obtained from the first author (ZH).

CMS was the primary outcome in this study, with a total score of 100. A higher score represents better function [32]. The scale assessed 4 domains, including pain level, ability to perform activities of daily living (ADL), AROM, and shoulder abduction strength. Participants state the severity of pain and limitations in ADL, and the remaining items are measured by the physiotherapist’s examination. Shoulder abduction strength was specifically assessed using the manual muscle testing technique [33]. Given that all participants had unilateral involvement, strength was evaluated concurrently on both the affected and unaffected sides. This side-to-side comparison during simultaneous abduction allowed for a relative grading of strength deficit, mitigating potential bias from interindividual variability in tester application.

UCLA Shoulder Scale is a system of functional assessment of surgical or conservative treatment of the shoulder with a total score of 35. It consists of pain, usable function of the upper extremity, ROM, and muscle strength of flexion, and patient satisfaction, with higher scores indicating better recovery. The scale focuses more on the impact of pain on daily life than the CMS. The muscle strength of flexion is also assessed using the manual muscle testing [33]. This outcome was not included in the trial registry and is reported in this manuscript as an exploratory outcome.

NRS uses a total of 11 numbers from 0 to 10 to show the severity of pain. After explaining the pain represented by each number, participants reported the highest level of pain experienced during the performance of daily activities in the week preceding the assessment time point, capturing the peak impact of pain on functional status. A score of 0 indicates no pain; 1‐3 is mild pain, and the pain does not interfere with sleep; 4‐6 is moderate pain that interferes with sleep; and 7‐10 is severe pain that prevents sleep [32].

Researcher 3 measured the shoulder ROM of flexion, abduction, external rotation, and internal rotation using a protractor in strictly standardized positions after excluding compensatory movements or interactions of neighboring joints [5,25].

Additional evaluated measures included treatment adherence, adverse events, and a system-derived performance metric. Adherence was tracked through a participant-maintained session log and verified during weekly clinic visits. All adverse events were actively monitored and documented throughout the study period. This included patient self-reports and standardized assessments using the Simulator Sickness Questionnaire [34] and the Virtual Reality Sickness Questionnaire [35]. Furthermore, the system generated a real-time score to improve the movement accuracy. This metric, henceforth termed system proficiency, provided intrasession biofeedback to guide movement correction and was not interpreted as a direct clinical measure of proprioception.

### Statistical Analysis

To determine statistical significance between 2 groups, continuous variables with a normal distribution were expressed as mean (SD), while non–normally distributed data were reported as median (IQR). Categorical data were presented as frequency (percentage) and compared using chi-square tests. For normally distributed data with homogeneity of variance, independent samples *t* tests were used for intergroup comparisons, while Welch *t* tests were used for heterogeneous variances. Friedman tests analyzed overall differences in non–normal data, with post hoc comparisons via the Conover-Iman test or Dunn multiple comparison test. Wilcoxon signed-rank tests assessed repeated measures, while Mann-Whitney *U* tests compared intergroup differences. As primary data violated normality assumptions, Scheirer-Ray-Hare tests (using an SPSS-validated alternative) evaluated main and interaction effects. Kruskal-Wallis H tests compared treatment efficacy across subgroups. Additionally, all analyses incorporated the Bonferroni correction. Data were analyzed using SPSS software (version 26.0; IBM Corp), with statistical significance set at a 2-tailed *P*<.05 and 95% CI.

### Ethical Considerations

This study was approved by the Medical Ethics Committee of Gongli Hospital, Pudong New Area, Shanghai, China (approval no. GLYY1s2024-031). Eligible participants were fully informed about the study’s purpose, procedures, potential risks, and benefits through verbal and written explanations. All participants provided written informed consent prior to enrollment. A unique number assigned to each participant served as an individual identifier, and all subsequent data analyses were performed exclusively on a deidentified dataset. Patients were not involved in the design or interpretation of the study results but contributed to data collection through their clinical outcomes. Although no participation compensation was provided, participants were entitled to receive appropriate alternative medical care—such as medication, shock wave therapy, or conventional rehabilitation exercises—if they experienced any adverse reactions (eg, allergy or dizziness) to the treatment protocol.

## Results

### Participant Characteristics

**Figure 2.**
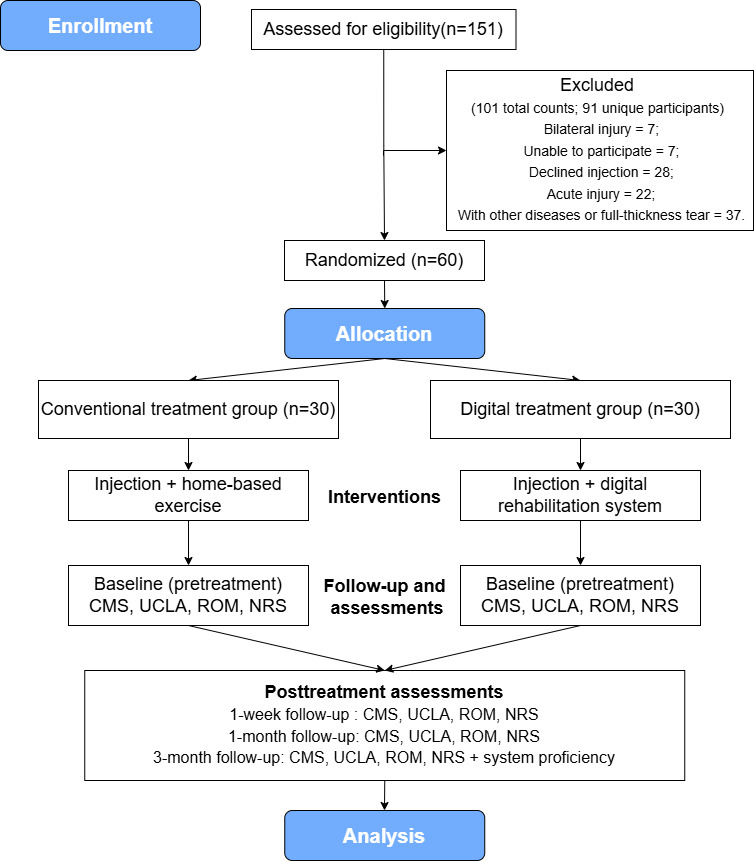
Flowchart of the randomized controlled trial in patients with chronic rotator cuff injury at Pudong Gongli Hospital, Shanghai University of Medicine & Health Sciences, Shanghai, China, 2025. Seven patients met multiple exclusion criteria; they are represented in different applicable categories but counted only once in the total (n=91). CMS: Constant-Murley Score; UCLA: University of California Los Angeles Shoulder Scale; ROM: range of motion; NRS: Numerical Rating Scale.

In this study, 151 participants were screened for eligibility, and a total of 60 participants met the inclusion criteria. The flowchart of participants in the trial, the screening, and trial processes are illustrated in Figure 2 [16]. 7 individuals were found to meet more than 1 exclusion criterion concurrently. One participant was both unable to participate and had a comorbid condition; 1 had a full-thickness tear, was in the acute injury phase, and declined the injection; 1 had a comorbid condition, was in the acute phase, and declined treatment; 2 had bilateral shoulder pain and comorbid conditions; 1 had bilateral shoulder pain and was in the acute phase; and 1 had bilateral shoulder pain, was in the acute phase, and declined treatment. The baseline characteristics of the 2 groups were well balanced, and statistical analysis revealed that no significant differences were observed between the 2 groups in terms of gender, age, disease duration, or side of injury (*P*>.05), and the results of the treatment were comparable (Table 1).

**Table 1.** Demographic and clinical characteristics of the 2 groups in a randomized controlled trial focusing on rotator cuff injuries at Pudong Gongli Hospital, Shanghai University of Medicine & Health Sciences, Shanghai, China, 2025.

Group	Gender		Injured side		Age (years), median (P25- P75)	Duration (months), median (P25-P75)
	Man, n (%)	Woman, n (%)	Left, n (%)	Right, n (%)		
Group C^a^	10 (33.3)	20 (66.7)	8 (26.7)	22 (73.3)	60 (52.25-69.25)	3 (3-6)
Group D^b^	9 (30)	21 (70)	9 (30)	21 (70)	63 (47.75-70)	3 (3-6)
*P* value	.781		>.99^c^		.976	.552

a Group C: conventional treatment group.

b Group D: digital treatment group.

c Fisher exact test was applied, resulting in *P*>.99. Pearson chi-square test yielded *P*=.774. All expected cell counts were >5 (minimum expected count = 8.50).

### Shoulder Functional Performance

**Figure 3.**
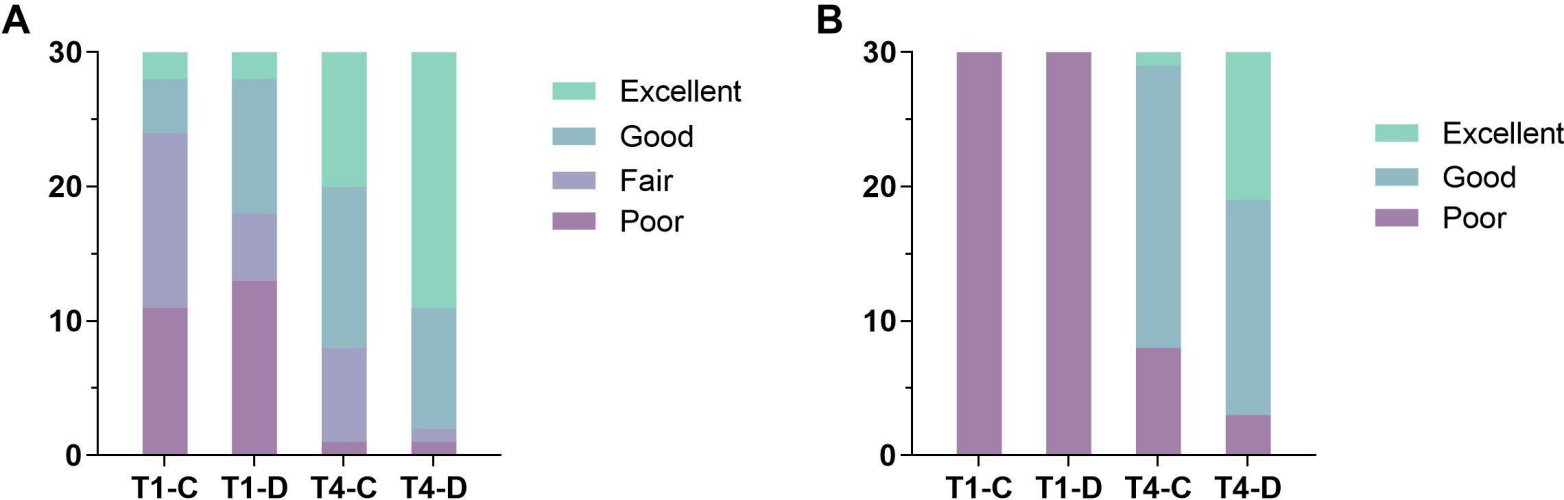
Proportion of participants with shoulder function scores at baseline and 3-month follow-up in a randomized controlled trial focusing on rotator cuff injuries at Pudong Gongli Hospital, Shanghai University of Medicine & Health Sciences, Shanghai, China, 2025. (A) Changes in the Constant-Murley Score for the 2 groups at baseline and 3-month follow-up (excellent: 90‐100, complete functional recovery; good: 80‐89, significant functional recovery; fair: 70‐79, partial improvement; and poor:<70, poor outcome). (B) Changes in the University of California Los Angeles Shoulder Scale for the 2 groups at baseline and 3-month follow-up (excellent, 34‐35; good, 29‐33; and poor,<29).

There was a significant time main effect (Kruskal-Wallis H=61.27, *P<*.001) and between-groups main effect (Kruskal-Wallis H=5.46, *P=*.019) for CMSs. For UCLA Shoulder Scale scores, a significant main effect of time was observed (Kruskal-Wallis H=116.91, *P<*.001). At T1, there was no statistically significant difference between the total CMS (*P=*.859) and UCLA Shoulder Scale scores (*P=*.405) of the 2 groups. At T4, the difference between the CMSs of the groups was even more significant (Hodges-Lehmann estimator [HLE] −6, 95% CI −10 to −2; *P=*.007). Compared with T1, both groups demonstrated significant CMS improvements at T4 (group C: median of difference [MD] 13.5, 95% CI 10.5-16.5, *P<*.001; group D: MD 19.0, 95% CI 14.5-22.5, *P<*.001). Notably, over half of group D (19/30, 63.3%) achieved “excellent” outcomes per Figure 3A substantially exceeding group C (10/30, 33.3%). UCLA Shoulder Scale scores similarly showed significant intergroup differences at T3 (HLE −2, 95% CI −3 to 0; *P=*.034) and T4 (HLE −2, 95% CI −4 to −1; *P=*.001). At T4, the number of participants rated as excellent in group D (11/30, 36.7%) exceeded that in group C (1/30, 3.3%) (Figure 3B). At T4, the improved scores of shoulder function were greater than the minimal clinically important difference (MCID) for CMS (7.2 points) and UCLA Shoulder Scale (3.3 points) (Table S1 in Multimedia Appendix 1) [36].

### Pain-Related Outcomes

Statistical analysis revealed significant main effects of both group (Kruskal-Wallis H=5.15, *P=*.023) and time (Kruskal-Wallis H=108.48, *P<*.001) on NRS scores. However, no significant group × time interaction was observed (Kruskal-Wallis H=7.38, *P*=.061). As shown in Table S1 in Multimedia Appendix 1, no statistically significant between-group differences in NRS scores were observed at T1 (*P*=.458). At T3, group D demonstrated a significant pain reduction from baseline (median 5, IQR 4‐6 to median 2, IQR 1‐2.25; *P<*.001; MD −3.5, 95% CI −3.5 to −3). This improvement further progressed at T4 (median 0.5, IQR 0‐2; *P<*.001; MD −4.0, 95% CI −4.5 to −3.5). Similarly, group C showed pain reduction at T3 (median 5, IQR 3‐6.25 to median 3, IQR 2‐3.25; *P*<.001; MD −2.0, 95% CI −2.5 to −1.5) and T4 (median 2, IQR 1-3; *P<*.001; MD −2.5, 95% CI −3 to −2). Both groups exceeded the MCID threshold of 1.37 points [37,38], indicating therapeutic relevance for both interventions. At T4, group D reported significantly lower pain intensity than group C (HLE 1, 95% CI 0-2; *P=*.002). These findings indicate enhanced pain-alleviating efficacy of group D compared with group C.

### Shoulder ROM

**Figure 4.**
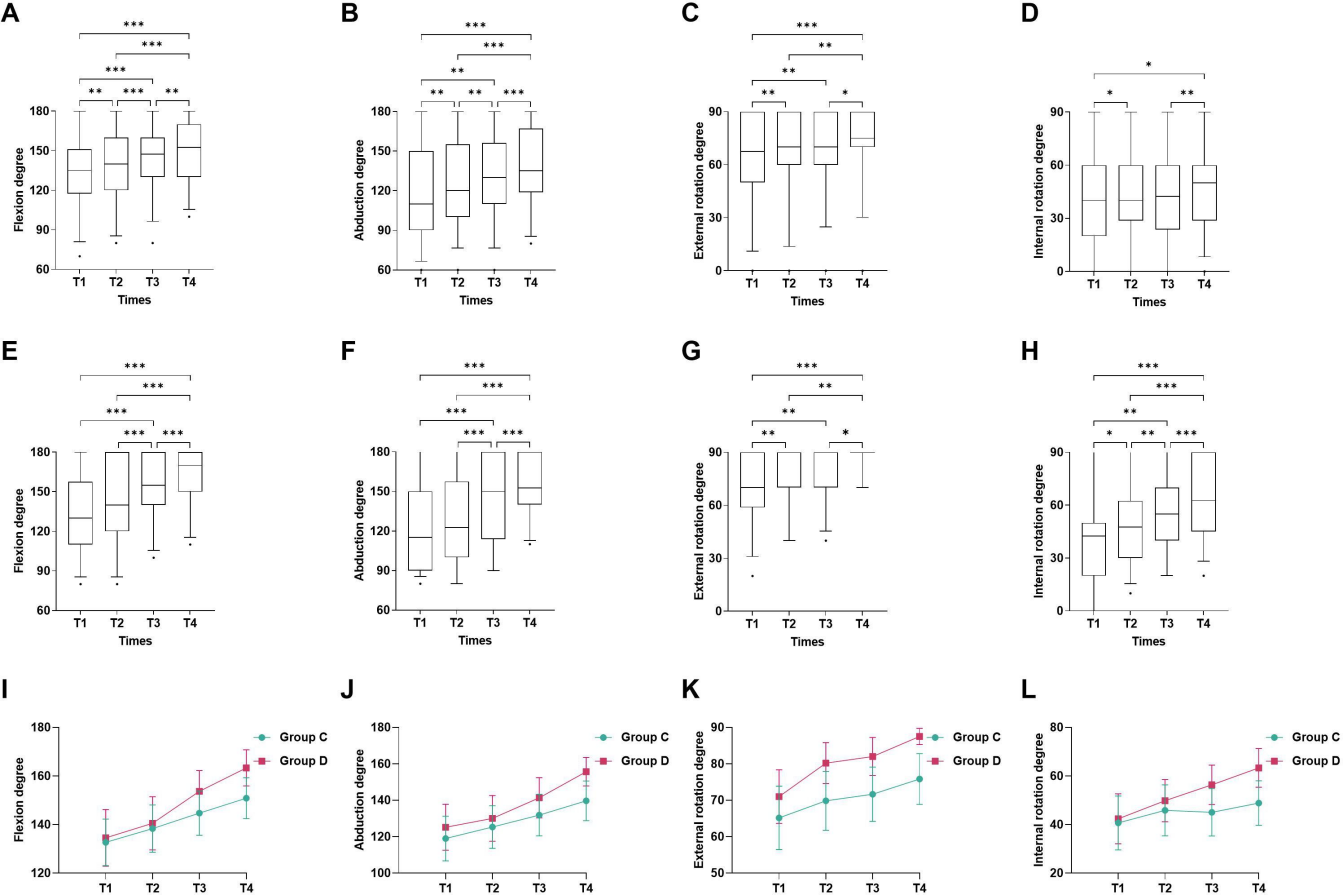
Changes within the range of motion of the 2 groups in a randomized controlled trial focusing on rotator cuff injuries at Pudong Gongli Hospital, Shanghai University of Medicine & Health Sciences, Shanghai, China, 2025. (A-D) Within-group differences in the range of motion for participants in the conventional treatment group; (E-H) within-group differences in the range of motion of the digital treatment group; and (I-L) comparisons between the 2 groups. *P<.05, **P<.01, ***P<.001.

The shoulder’s 3 rotational degrees of freedom contribute to heterogeneous motion limitations post-RCI. This study conducted analyses on ROM in 4 movement directions: flexion, abduction, external rotation, and internal rotation. Significant main effects of time were observed for all 4 ROM directions, but no time × group interactions emerged. Group effects existed for all directions except flexion. Baseline data showed no significant between-group differences in any plane (Table S1 in Multimedia Appendix 1). At T2 and T3, only the ROM of external rotation demonstrated significant intergroup differences (Figure 4I-L). By T4, group D exhibited significantly higher median ROM than group C in flexion (HLE −10, 95% CI −25 to 0; *P=*.023), abduction (HLE −15, 95% CI −30 to 0; *P=*.034), external rotation (HLE −5, 95% CI −20 to 0; *P=*.001), and internal rotation (HLE −15, 95% CI −30 to 0; *P=*.035) (Figure 4A-H). All directions showed increased ROM at T4 versus T1 (Figure 4), with maximal gains in abduction. However, transient ROM reductions occurred during training.

### Other Findings

All patients fully complied with the prescribed protocols, and no deviations occurred during the study. No adverse events were reported or identified through patient logs and standardized questionnaires throughout the study period. The monitored outcomes specifically included, but were not limited to, muscle atrophy, abnormal blood glucose, allergic reactions, and dizziness. No participants required rescue analgesic medication during the study period. System proficiency, collected as process feedback during training, was treated as a nonclinical metric. It is presented in the final subsection of the “Results” and analyzed in the “Discussion” section.

### Subgroup Analysis

The distribution of participants across these subgroups was comparable between group C and group D. As presented in Table 2 and Tables S2-S4 in Multimedia Appendix 1, the baseline demographic and clinical characteristics were well balanced across all 3 subgroups within each treatment group. This balance in baseline profiles confirms that any observed differences in treatment outcomes among the subgroups are unlikely to be attributable to preexisting disparities in these key variables.

**Table 2.** Statistical comparison of characteristics across the subgroups stratified by baseline function within each intervention group in a randomized controlled trial focusing on rotator cuff injuries at Pudong Gongli Hospital, Shanghai University of Medicine & Health Sciences, Shanghai, China, 2025.

Subgroup	Gender		Injured side		Age (years), median (P25-P75)	Duration (months), median (P25-P75)
	Man, n (%)	Woman, n (%)	Left, n (%)	Right, n (%)		
1						
Group C (n=7)^a^	1 (14.3)	6 (85.7)	1 (14.3)	6 (85.7)	64 (53-68)	6 (3-12)
Group D (n=9)^b^	3 (33.3)	6 (66.7)	1 (11.1)	8 (88.9)	62 (46.5-69.5)	3 (3-8)
*P* value	.372		.849		.916	.350
2						
Group C (n=9)^a^	5 (55.6)	4 (44.4)	2 (22.2)	7 (77.8)	60 (49-71.5)	3 (3-3.5)
Group D (n=8)^b^	3 (37.5)	5 (62.5)	3 (37.5)	5 (62.5)	57.5 (45-69)	3 (3-3.75)
*P* value	.455		.490		.586	.432
3						
Group C (n=14)^a^	4 (28.6)	10 (71.4)	5 (35.7)	9 (64.3)	60 (53-69.25)	4 (3-6)
Group D (n=13)^b^	3 (23.1)	10 (76.9)	5 (38.5)	8 (61.5)	64 (55-70.5)	3 (3-6)
*P* value	.744		.883		.451	.979

a C: conventional treatment group.

b D: digital treatment group.

After adjusting for baseline differences using ANCOVA, the pattern of therapeutic effects differed between group C and group D. Within group C, therapeutic effects were largely similar across the 3 subgroups, with the exception of shoulder abduction. For abduction, the homogeneity of variance assumption was violated (Levene test, *F*_2, 27_=3.681; *P*=.039). Therefore, the Games-Howell post hoc test, which does not assume equal variances, was used. It revealed that the improvement in subgroup 1 was significantly greater than in both subgroup 2 (mean difference 56.49, 95% CI 38.33-74.66; *P*<.001) and subgroup 3 (mean difference 36.57, 95% CI 16.83-56.31; *P*<.001).

In contrast, within group D, significant subgroup differences were observed across multiple outcomes. For internal rotation, where variances were homogeneous (Levene test, *F*_2, 27_=0.845; *P*=.441), a standard 1-way ANCOVA revealed a significant main effect of subgroup (*F*_2, 26_=10.848; *P*<.001, η²=0.455). Post hoc pairwise comparisons (Bonferroni-corrected) confirmed that subgroup 1 improved significantly more than both subgroup 2 (mean difference 32.16, 95% CI 13.28-51.04; *P*=.001) and subgroup 3 (mean difference 24.48, 95% CI 8.49-40.47; *P*=.002). For shoulder flexion (Levene *F*_2, 27_=7.333; *P*=.003), UCLA Shoulder Scale score (Levene *F*_2, 27_=4.911; *P*=.015), and shoulder abduction (Levene *F*_2, 27_=3.776; *P*=.036), the variance homogeneity assumption was violated. Consequently, the Games-Howell post hoc tests were applied. These analyses indicated significant subgroup differences for all 3 measures (all *P*<.05, as evidenced by the subsequent pairwise comparisons). Specifically, subgroup 1 showed superior improvement to both subgroup 2 and subgroup 3 in flexion, UCLA Shoulder Scale score, and abduction (all *P*<.05, in Table S5 in Multimedia Appendix 1). Notably, for abduction, a further significant difference was found, with subgroup 3 improving more than subgroup 2 (mean difference 23.89, 95% CI 5.88-41.90; *P*=.010), establishing a clear improvement gradient: subgroup 1 > subgroup 3 > subgroup 2.

**Figure 5.**
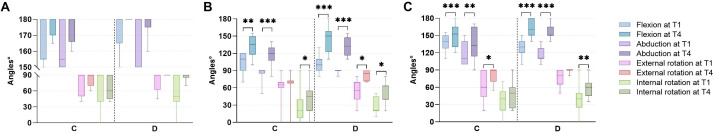
Efficacy of conventional and digital treatments in each subgroup in a randomized controlled trial focusing on rotator cuff injuries at Pudong Gongli Hospital, Shanghai University of Medicine & Health Sciences, Shanghai, China, 2025. (A) Range of motion of subgroup 1; (B) range of motion of subgroup 2; and (C) range of motion of subgroup 3. *P<.05, **P<.01, ***P<.001.

Significant improvements in CMS, UCLA Shoulder Scale, and NRS in global mixed-rank and within-group changes were observed in all subgroups at T4 compared with T1. Subgroups 2 and 3 additionally demonstrated a statistical difference in shoulder ROM (Figure 5A-C and Table S2-S4 in Multimedia Appendix 1), suggesting that combined injection-rehabilitation therapy primarily enhances mobility in restricted ones.

### System Proficiency

At T4, group D demonstrated higher median (P25-P75) scores than group C in flexion (93, 88.75-95 vs 87.5, 77.75-92.25; *P*<.001), abduction (86.5, 81.5-89 vs 84, 70-86.75; *P*=.048), external rotation (88.5, 85.75-92.25 vs 79, 52-90; *P*=.009), and internal rotation (89, 83-92 vs 66.5, 42.25-86; *P*<.001). Subgroup analyses offered further insight. Within subgroup 2, group D demonstrated superior median (P25-P75) scores than group C in external rotation (88, 83.25-90.75 vs 61, 45.5-80.5; HLE −28, 95% CI −43 to 0; *P*=.036) and internal rotation (mean 88.75, SD 4.496 vs mean 50.67, SD 25.598; mean difference −38.08, 95% CI −57.88 to −18.29; *t*_8.553_=−4.39; *P*=.002).

In subgroup 3, group D demonstrated greater proficiency (median, P25-P75) than group C in flexion (93, 89-96 vs 84.5, 76.75-92; HLE −9, 95% CI −15 to −2; *P*=.003), abduction (88, 85.5-90 vs 82, 66.5-89.25; HLE −7, 95% CI −19 to −1; *P*=.019), and internal rotation (85, 78-91.5 vs 73.5, 45-83; HLE −11, 95% CI −38 to 0; *P*=.043).

## Discussion

### Overview and Background

RCI refers to pathological changes in the supraspinatus, infraspinatus, teres minor, or subscapularis tendons, with the supraspinatus most commonly affected [39]. Although passive ROM theoretically remains intact in these patients, clinical observations reveal that pain and protective neuromuscular mechanisms following tendon injury frequently result in limitations in both AROM and passive ROM. Home-based rehabilitation exhibits inherent limitations in standardizing exercise parameters under the FITT (Frequency, Intensity, Time, and Type) framework, often resulting in inconsistent recovery outcomes. Furthermore, patients with chronic diseases are consistently challenged by persistent pain, movement restrictions, and insufficient professional guidance, which collectively compromise exercise adherence and rehabilitation efficacy. Despite the rapid emergence of DTx, their application remains predominantly focused on postoperative rehabilitation [14], with evidence-based research on nonsurgical shoulder interventions still in exploratory phases. This study innovatively combines injection with digital rehabilitation modalities, providing a systematic evaluation of this protocol’s clinical effectiveness in the conservative management of CRCI.

### Research Findings and Comparison With Prior Work

The Digital Rehabilitation System was customized based on the kinematic characteristics of CRCI. Consistent with prior findings [40], the digital protocol demonstrated superior pain reduction and functional recovery. The improvements of CMS, UCLA Shoulder Scale, and NRS exceeded their MCIDs [36-38], underscoring the critical role of real-time visualization and biofeedback mechanisms [11]. While the studies by AlHossan et al [41] and Pak et al [13] concluded that digital and conventional exercise yield comparable outcomes for chronic shoulder pain rehabilitation, our study extends these findings by demonstrating superior efficacy in a distinct, nonoperative patient population. This supports the broader applicability and integration of digital technology into shoulder rehabilitation protocols. And discrepancies between our findings and the negative conclusions reported by Shim et al [11] may stem from population heterogeneity, intervention protocol variations, and differential sensitivity of outcome measures.

Postintervention analysis revealed significant between-group differences favoring group D in pain (NRS, *P*=.002), shoulder function (CMS, *P*=.007; UCLA Shoulder Scale, *P*=.001), and AROM for flexion (*P*=.023), abduction (*P*=.034), external rotation (*P*=.001), and internal rotation (*P*=.035). Notably, 6 participants (4 in group C, 2 in group D) exhibited a reduction in ROM during the treatment period, which increased data heterogeneity of detecting significant between-group differences.

The finding of greater absolute rotation ROM in group D posttreatment may primarily reflect restored tissue extensibility. However, neuromuscular coordination issues may still remain unresolved. Compensatory patterns, such as scapulothoracic dyskinesis [42] and inherent rotational biomechanical constraints [43], can drive avoidance of true rotational movements, thereby perpetuating deficits [44]. Furthermore, patients’ predominant reliance on flexion during ADL may create “blind zones” for rotational control in self-directed rehabilitation. If unaddressed, these issues can contribute to sustained muscle hypertonicity, strain on nontarget muscles, and increased joint stress, ultimately impeding functional recovery. Patients with severe capsular adhesions or pronounced neuromuscular dysfunction may therefore require integrated, adjunctive treatments to fully capitalize on the benefits of exercise therapy.

### Scientific Rationale for the Combined Intervention

Rehabilitation exercises promote collagen synthesis and tendon strengthening. The system’s algorithms address the corrective feedback delays inherent in conventional methods via real-time biofeedback, while motion visualization enhances sensorimotor cortical reorganization and neuromuscular control. The corticosteroid component of the injection suppresses synovitis and proinflammatory cytokines [5,39], rapidly alleviating pain and edema to remove barriers to subsequent training. Additionally, local anesthetics modulate nociceptive signaling and collagen remodeling to restore tendon homeostasis [3]. Integrating injection with digital rehabilitation ensures biomechanically optimized training postures, thereby enhancing functional outcomes while minimizing reinjury risks.

### Ceiling Effects in Subgroup Analysis

First, a methodological ceiling effect was inherent in the composition of subgroup 1. This subgroup was specifically defined by a combined shoulder flexion and abduction ROM of ≥150° for both movements at baseline. This threshold represents a functional level near the upper limit of normal daily requirements. Consequently, participants in this subgroup began the study at a functional ceiling, leaving minimal room for measurable improvement on the ROM scales themselves. The primary rationale for creating this distinct subgroup was to isolate and analyze the treatment response in participants whose primary complaint was pain without substantial functional impairment. Including these high-functioning individuals in the overall analysis would have diluted or obscured the detection of true functional gains in participants with more significant baseline limitations (subgroups 2 and 3), as their near-maximal scores would disproportionately influence the group means or medians.

Second, a notable observation pertains to the analysis of internal rotation in subgroup 1 at the T2 time point. The Mann-Whitney *U* test between group C and group D yielded *P*>.99 (Table S2 in Multimedia Appendix 1). Crucially, this was not due to identical raw data but due to identical rank sums (eg, both groups having a median rank of 8.5). This occurs because when the vast majority of participants in a subgroup are clustered at the very top of the measurement scale, the variance in raw scores becomes compressed. The rank-based test then loses its discriminative power, as small, clinically irrelevant differences near the ceiling are insufficient to generate a significant rank separation. Thus, the *P* value of 1.000 is a statistical artifact of the measurement ceiling, indicating that the tool was insensitive to differences at this high functional level at that specific time point, not necessarily the absence of any differential treatment effect.

### Heterogeneous Treatment Response Based on Baseline Function

A key finding from the subgroup analysis was the differential efficacy of group D across patients with varying baseline function. While the unadjusted improvement magnitudes (Δ) of flexion, abduction, and external rotation suggested that subgroup 2 gained the most (Tables S2-S4 in Multimedia Appendix 1), the ANCOVA—which statistically equates baseline scores—revealed that subgroup 1 achieved a superior adjusted treatment effect. It is important to note that this ANCOVA is a within-arm analysis and does not directly establish differential between-treatment efficacy. This demonstrates that the apparent advantage of the severely impaired subgroup was likely confounded by its greater potential range for absolute improvement. After accounting for this baseline disparity, the analysis indicates that the intervention was particularly effective in further optimizing function in those who started with no or only mild limitations. Given the small per-subgroup sample sizes (n=7-14), statistical power for between-group comparisons was limited. Accordingly, these subgroup analyses should be interpreted as exploratory and hypothesis-generating.

This pattern suggests a heterogeneous treatment response, potentially mediated by the patient’s initial functional capacity. Patients with no or milder impairments may possess a sufficient residual movement repertoire and neuromuscular control to more fully exploit and integrate the precise, feedback-driven training. Consequently, the digital rehabilitation protocol appears especially potent as an optimization tool for patients at a higher functional baseline, enabling them to achieve near-normal performance levels, rather than as a primary restorative intervention for those with severe deficits.

### System Proficiency and Its Clinical Implications

The digital rehabilitation system used in this study incorporated a real-time score derived from a weighted composite of 4 kinematic parameters: trunk inclination, target-plane angle, compensatory motion in nontarget planes, and postural alignment during exercise. This score provided immediate biofeedback to optimize movement quality. However, as group C did not train with this system, the score may reflect system-specific proficiency—familiarity with the device and its feedback—rather than a pure, transferable measure of proprioception. Consequently, it was not interpreted as a primary clinical outcome.

### Impact of Inclusion Criterion Deviation on Study Findings

A discrepancy in the pain severity inclusion criterion occurred (registry: NRS score ≥4; final analysis: NRS score ≥3) because NRS score 3 represents a clinical transition zone between mild and moderate pain, where patients often experience functional impairment despite meeting the numerical definition of mild pain, making the distinction between scores 3 and 4 difficult in practice. To assess whether this adjustment introduced bias, a series of analyses was conducted. First, a comparison between patients with NRS score 3 (n=10) and NRS score ≥4 (n=50) showed no significant differences in baseline characteristics (all *P*>.05; Table S6 in Multimedia Appendix 1), indicating that the 10 patients were comparable with the rest of the cohort. Second, after excluding these 10 patients, group C and group D remained well balanced in baseline characteristics (all *P*>.05; Table S7 in Multimedia Appendix 1). Finally, sensitivity analysis excluding patients with NRS score 3 yielded results consistent with the full analysis (Table S8 vs Table S1 in Multimedia Appendix 1), with the direction and significance of treatment effects at T4 remaining unchanged. These findings demonstrate that the inclusion of patients with NRS score 3 did not introduce bias and that our conclusions are robust.

It is noteworthy that in the sensitivity analysis cohort, 8 of the 10 excluded participants were from group C, resulting in unequal final group sizes (group C: n=22; group D: n=28). This reflects the randomness of the initial allocation rather than a systematic bias, as the baseline characteristics remained balanced. While unequal group sizes can influence statistical power, the consistency of the treatment effects in both the full and sensitivity analyses supports the robustness of our primary conclusions regarding the efficacy of the digital intervention.

### Limitations and Future Work

This study has several limitations that should be acknowledged. First, as a pilot study with a primary focus on understanding initial therapeutic effects, the follow-up period was limited to 3 months. While sufficient to assess the short-term efficacy and safety of the intervention, it may not capture the long-term interaction between the waning anti-inflammatory effects of corticosteroids and the sustainability of rehabilitation gains. Although the pharmacological half-life of corticosteroids is short (eg, 36‐54 hours for compound betamethasone), their biological effects through genomic mechanisms persist for a much longer duration. Future studies should incorporate extended follow-up assessments at 6 and 12 months and implement structured retention strategies, such as participant incentives, which would be crucial to ensure adherence in longer-term trials.

Second, the single-center design and the modest sample size (N=60) limit the generalizability of the findings. While informative for initial efficacy signals, the statistical power for subgroup analyses was constrained, potentially affecting the robustness of these comparisons. To enhance external validity and confirm the results, a subsequent multicenter randomized controlled trial (in collaboration with the community or other hospitals) with a larger cohort is warranted.

Third, the current system’s training paradigm may be relatively simple for participants presenting with pain alone, potentially influencing the average system proficiency scores. Furthermore, the protocol lacked a dedicated module for proprioceptive training. Future iterations of the rehabilitation system should integrate modular and comprehensive training strategies, including randomized angle proprioception drills, to better address neuromuscular control deficits and potentially improve overall outcomes.

Fourth, concerning blinding, although all participants were exposed to the VR sessions to standardize initial exposure, complete participant blinding was not feasible due to the distinct active interventions (ie, only group D received motion capture–based training). This could have introduced performance bias or a novelty effect. A future design could use a modified “digital sham” control, where both groups interact with the digital platform, but group C receives a version that provides feedback only during assessment periods rather than as real-time training guidance, thereby better controlling for these nonspecific effects.

### Conclusions

This study demonstrates that combining ultrasound-guided steroid-anesthetic injections with subsequent Digital Rehabilitation System training provides dual therapeutic benefits for CRCI. It effectively reduces pain severity while improving shoulder joint ROM and shoulder function scores. This integrated approach holds significant clinical implications, with particularly pronounced benefits observed in subgroups exhibiting no or mild shoulder mobility restrictions.

## Supplementary material

10.2196/79494 Multimedia Appendix 1 Supplementary material containing primary analysis, subgroup analyses, sensitivity analyses, and supporting data.

10.2196/79494 Checklist 1 CONSORT-HEALTH (V 1.6.1) checklist.

## Abbreviations

ADL: activities of daily living
AROM: active range of motion
CMS: Constant-Murley Score
CONSORT-EHEALTH: Consolidated Standards of Reporting Trials of Electronic and Mobile Health Applications and Online Telehealth
CRCI: chronic rotator cuff injury
DTx: digital therapeutics
FITT: Frequency, Intensity, Time, and Type
HLE: Hodges-Lehmann estimator
MCID: minimal clinically important difference
MCS: Motion Capture subsystem
MD: median of difference
NRS: Numerical Rating Scale
RCI: rotator cuff injury
ROM: range of motion
T1: baseline
T2: 1-week follow-up
T3: 1-month follow-up
T4: 3-month follow-up
UCLA: University of California Los Angeles
VR: virtual reality
VRS: Virtual Reality Education subsystem
