# Supplementary material for: Digital Rehabilitation Following Ultrasound-Guided Injection for Chronic Rotator Cuff Injury: Randomized Controlled Trial
Source: J Med Internet Res. 2026 Jun 10;28:e79494. doi: 10.2196/79494 (PMC13251545; doi:10.2196/79494)
Supplement: Multimedia Appendix 1 [file jmir-v28-e79494-s001.doc]

Multimedia Appendix 1

Table S1. Shoulder performance in the Conventional Treatment Group and the Digital Treatment Group in a randomized controlled trial focusing on rotator cuff injuries at Pudong Gongli Hospital, Shanghai University of Medicine & Health Sciences, Shanghai, China, 2025.

|  | Groups | T1 | T2 | T3 | T4 | Δ |
| --- | --- | --- | --- | --- | --- | --- |
| CMS | C | 73(66,78) | 79(70.75,82.5)^c^ | 81.5(75.25,86.25)^d^ | 86(78.75,91.25)^d^ | 13.53±7.399 |
|  | D | 73(58.5,83.25) | 79(72,88.25) | 88(81.5,91.25)^d^ | 91(85.75,96.5)^d^ | 18.9±9.267 |
|  | *P* value | .859 | .906 | .029 | .007 |  |
| UCLA | C | 21(17.75,23.25) | 28(25,30.25)^d^ | 29(25,31)^d^ | 30.5(28,33)^d^ | 9.8±2.657 |
|  | D | 19(15.75,23) | 27(24,32)^c^ | 31.5(28,33)^d^ | 33(31,35)^d^ | 12.8±3.418 |
|  | *P* value | .405^a^ | .941 | .034 | .001 |  |
| NRS | C | 5(3,6.25) | 3(2.75,5)^b^ | 3(2,3.25)^d^ | 2(1,3)^d^ | 2.67±1.422 |
|  | D | 5(4,6) | 4(2,4)^b^ | 2(1,2.25)^d^ | 0.5(0,2)^d^ | 4.03±1.402 |
|  | *P* value | .458 | .856 | .001 | .002 |  |
| Flexion | C | 135(117.5,151.25) | 140(120,160) | 147.5(130,160)^d^ | 152.5(130,170)^d^ | 18.17±14.049 |
|  | D | 130(110,157.5) | 140(120,180) | 155(140,180)^d^ | 170(150,180)^d^ | 28.83±22.464 |
|  | *P* value | .923 | .876 | .165 | .023 |  |
| Abduction | C | 110(90,150) | 120(100,155) | 130(110,156.25)^c^ | 135(118.75,167)^d^ | 20.7±20.194 |
|  | D | 115(90,150) | 122.5(100,157.5) | 150(113.75,180)^c^ | 152.5(140,180)^d^ | 30.5±22.489 |
|  | *P* value | .550 | .666 | .280 | .034 |  |
| External Rotation | C | 67.5(50,90) | 70(60,90) | 70(60,90) | 75(70,90)^c^ | 10.67±15.687 |
|  | D | 70(58.75,90) | 90(70,90) | 90(70,90) | 90(90,90)^d^ | 16.5±16.72 |
|  | *P* value | .354 | .030 | .018 | .001 |  |
| Internal Rotation | C | 40(20,60) | 40(28.75,60) | 42.5(23.75,60) | 50(28.75,60)^d^ | 8.17±18.122 |
|  | D | 42.5(20,50) | 47.5(30,62.5) | 55(40,70)^b^ | 62.5(45,90)^d^ | 21±23.022 |
|  | *P* value | .771 | .512 | .074^a^ | .035 |  |

^a^ Data are normally distributed. ^b^ Post hoc pairwise comparisons versus baseline *P<*.05. ^c^ Post hoc pairwise comparisons versus baseline *P<*.01. ^d^ Post hoc pairwise comparisons versus baseline *P<*.001.

T1, baseline assessment; T2, 1-week follow-up; T3, 1-month follow-up; T4, 3-month follow-up; Δ, the improvement value between baseline and 3-month follow-up; C, the Conventional Treatment Group (n=30); D, the Digital Treatment Group (n=30); CMS, the Constant-Murley Score; UCLA, the University of California Los Angeles Shoulder Scale; NRS, Numerical Rating Scale.

Table S2. Shoulder performance in the subgroup with no or mild limitation in a randomized controlled trial focusing on rotator cuff injuries at Pudong Gongli Hospital, Shanghai University of Medicine & Health Sciences, Shanghai, China, 2025.

| Subgroup 1 | | T1 | T2 | T3 | T4 | Δ^e^ |
| --- | --- | --- | --- | --- | --- | --- |
| CMS | C | 80(78,90) | 87(82,93) | 91(86,94) | 93(89,96)^c^ | 10.71±8.939 |
|  | D | 86(83.5,89.5) | 91(88.5,93) | 93(91,97.5)^c^ | 98(93.5,100)^d^ | 10.33±3.708 |
|  | *P* value | .079^a^ | .168^a^ | .08^a^ | .028 |  |
| UCLA | C | 24(21,26) | 31(30,33) | 31(30,33) | 33(33,33)^c^ | 9.43±2.37 |
|  | D | 25(22,27.5) | 33(31.5,33) | 33(33,33.5)^c^ | 35(33.5,35)^d^ | 9.89±3.06 |
|  | *P* value | .497^a^ | .212 | .011 | .018 |  |
| NRS | C | 4(3,7) | 3(2,4) | 2(2,4) | 1(0,2)^c^ | 3.14±1.574 |
|  | D | 4(4,5) | 3(2,4) | 1(0.5,2)^b^ | 0(0,1)^d^ | 3.89±1.616 |
|  | *P* value | .502 | .592^a^ | .061^a^ | .127 |  |
| Flexion | C | 155(155,180) | 160(155,180) | 180(160,180) | 180(170,180) | 12.86±10.746 |
|  | D | 180(165,180) | 180(180,180) | 180(180,180) | 180(180,180) | 6.67±13.229 |
|  | *P* value | .177 | .106 | .235 | .036 |  |
| Abduction | C | 155(150,180) | 160(155,180) | 170(160,180) | 180(166,180) | 10.86±12.786 |
|  | D | 180(150,180) | 180(165,180) | 180(165,180) | 180(175,180) | 6.67±11.18 |
|  | *P* value | .388 | .219 | .22 | .402 |  |
| External Rotation | C | 90(50,90) | 90(60,90) | 90(60,90) | 90(70,90) | 5.71±9.759 |
|  | D | 90(62.5,90) | 90(90,90) | 90(90,90) | 90(90,90) | 12.22±17.341 |
|  | *P* value | .808 | .098 | .097 | .098 |  |
| Internal Rotation | C | 90(40,90) | 90(40,90) | 60(40,90) | 60(45,90) | 0.71±23.171 |
|  | D | 50(40,90) | 60(55,90) | 80(65,90) | 90(85,90) | 26.67±32.016 |
|  | *P* value | .65 | >.99 | .27^a^ | .068 |  |

^a^ Data are normally distributed. ^b^ Post hoc pairwise comparisons versus baseline *P<*.05. ^c^ Post hoc pairwise comparisons versus baseline *P<*.01. ^d^ Post hoc pairwise comparisons versus baseline *P<*.001. ^e^ Δ values represent the unadjusted improvement within each subgroup. They should not be used for direct statistical comparison between subgroups due to baseline differences.

T1, baseline assessment; T2, 1-week follow-up; T3, 1-month follow-up; T4, 3-month follow-up; C, the Conventional Treatment Group (n=7); D, the Digital Treatment Group (n=9); CMS, the Constant-Murley Score; UCLA, the University of California Los Angeles Shoulder Scale; NRS, Numerical Rating Scale.

Table S3. Shoulder performance in the subgroup with severe limitation in a randomized controlled trial focusing on rotator cuff injuries at Pudong Gongli Hospital, Shanghai University of Medicine & Health Sciences, Shanghai, China, 2025.

| Subgroup 2 | | T1 | T2 | T3 | T4 | Δ^e^ |
| --- | --- | --- | --- | --- | --- | --- |
| CMS | C | 66(54,73) | 70(64,76.5) | 76(71,80.5)^c^ | 83(74.5,84)^d^ | 16.56±7.333 |
|  | D | 57.5(50.5,65.75) | 69(52.25,77.25) | 79.5(63.5,82.75)^b^ | 83.5(74,90.5)^d^ | 23.63±4.658 |
|  | *P* value | .246 | .846 | .441 | .599^a^ |  |
| UCLA | C | 17(14.5,18.5) | 26(19,28) | 28(25,29.5)^b^ | 29(26,30.5)^d^ | 11.89±2.667 |
|  | D | 14.5(12.5,18) | 24.5(17.5,26.5) | 28(23,30)^b^ | 31(24,33)^d^ | 13.88±2.167 |
|  | *P* value | .663^a^ | .499 | .845 | .556^a^ |  |
| NRS | C | 5(4,6.5) | 4(3,6) | 3(2,4) | 3(1.5,3.5)^c^ | 2.56±1.509 |
|  | D | 5(4,6.75) | 3.5(2,4.75) | 2(1.25,2.75)^c^ | 1(0,2)d | 4±1.309 |
|  | *P* value | .852^a^ | .427^a^ | .147 | .056 |  |
| Flexion | C | 110(90,120) | 120(95,130) | 130(115,130)^b^ | 135(117.5,150)^c^ | 27.78±17.52 |
|  | D | 100(90,110) | 120(92.5,128.75) | 132.5(112.5,140)^b^ | 150(122.5,158.75)^d^ | 43.13±23.136 |
|  | *P* value | .697^a^ | .792^a^ | .46 | .249^a^ |  |
| Abduction | C | 90(85,90) | 90(90,105) | 110(90,122.5)^b^ | 120(107.5,130)^d^ | 32.78±13.255 |
|  | D | 90(90,90) | 92.5(82.5,100) | 110(92.5,126.25)^c^ | 132.5(116.25,147.5)^d^ | 43.13±14.623 |
|  | *P* value | .562 | .829^a^ | .637^a^ | .1^a^ |  |
| External Rotation | C | 65(60,70) | 70(62.5,70) | 70(67.5,70) | 70(67.5,72.5) | 3.89±6.009 |
|  | D | 55(40,70) | 70(45,77.5) | 70(52.5,77.5) | 85(71.25,90)^b^ | 28.13±16.89 |
|  | *P* value | .656 | .842 | .876 | .023 |  |
| Internal Rotation | C | 20(7.5,40) | 30(12.5,50) | 40(17.5,55) | 45(20,55)^b^ | 13.33±13.463 |
|  | D | 20(20,45) | 30(20,40) | 37.5(21.25,57.5) | 40(40,63.75)^b^ | 19.38±18.213 |
|  | *P* value | .582 | .684^a^ | .924^a^ | .587^a^ |  |

^a^ Data are normally distributed. ^b^ Post hoc pairwise comparisons versus baseline *P<*.05. ^c^ Post hoc pairwise comparisons versus baseline *P<*.01. ^d^ Post hoc pairwise comparisons versus baseline *P<*.001. ^e^ Δ values represent the unadjusted improvement within each subgroup. They should not be used for direct statistical comparison between subgroups due to baseline differences.

T1, baseline assessment; T2, 1-week follow-up; T3, 1-month follow-up; T4, 3-month follow-up; C, the Conventional Treatment Group (n=9); D, the Digital Treatment Group (n=8); CMS, the Constant-Murley Score; UCLA, the University of California Los Angeles Shoulder Scale; NRS, Numerical Rating Scale.

Table S4. Shoulder performance in the subgroup with moderate limitation in a randomized controlled trial focusing on rotator cuff injuries at Pudong Gongli Hospital, Shanghai University of Medicine & Health Sciences, Shanghai, China, 2025.

| Subgroup 3 | | T1 | T2 | T3 | T4 | Δ^e^ |
| --- | --- | --- | --- | --- | --- | --- |
| CMS | C | 73(66.75,78) | 79(76,82.5) | 83(75.25,85.25) | 86.5(78.75,90)^d^ | 13±6.421 |
|  | D | 71(59,79) | 76(71,81) | 88(81,89.5)^d^ | 91(87.5,95)^d^ | 21.92±10.291 |
|  | *P* value | .32^a^ | .263 | .059^a^ | .017^a^ |  |
| UCLA | C | 21(19.5,23.5) | 28.5(25,30) | 28.5(25,30.25) | 30(28,31.5)^d^ | 8.64±2.061 |
|  | D | 19(16,20.5) | 26(23.5,29) | 31(28,32)^c^ | 33(31,34)^d^ | 14.15±3.184 |
|  | *P* value | .028^a^ | .251 | .072^a^ | .003^a^ |  |
| NRS | C | 5(3,5.5) | 3(2,5) | 3(2,3.5) | 2(1.75,3)^d^ | 2.5±1.345 |
|  | D | 5(4.5,6.5) | 4(3,4.5) | 2(1,3)^d^ | 1(0,2)^d^ | 4.15±1.405 |
|  | *P* value | .249 | .357 | .021 | .052 |  |
| Flexion | C | 140(127.5,150) | 140(120,160) | 150(128.75,160)^b^ | 152.5(130,165)^d^ | 14.64±10.278 |
|  | D | 130(120,140) | 140(120,145) | 155(140,157.5)^d^ | 160(150,180)^d^ | 35.38±15.473 |
|  | *P* value | .138^a^ | .333^a^ | .406 | .116 |  |
| Abduction | C | 110(100,140) | 122.5(110,143.75) | 127.5(110,150) | 132.5(113.75,165)^c^ | 17.86±23.755 |
|  | D | 110(110,127.5) | 120(110,140) | 130(122.5,150)^d^ | 150(150,165)^d^ | 39.23±19.984 |
|  | *P* value | .96 | .604^a^ | .362^a^ | .086 |  |
| External Rotation | C | 60(43.75,90) | 75(52.5,90) | 70(58.75,90) | 90(70,90)^b^ | 17.5±19.685 |
|  | D | 80(65,90) | 90(70,90) | 90(90,90) | 90(90,90)^c^ | 12.31±13.634 |
|  | *P* value | .076 | .056 | .02 | .027 |  |
| Internal Rotation | C | 40(20,52.5) | 42.5(33.75,52.5) | 37.5(23.75,52.5) | 50(23.75,60) | 8.57±18.02 |
|  | D | 40(25,50) | 45(30,60) | 50(40,65) | 60(45,67.5) | 18.08±19.207 |
|  | *P* value | .786^a^ | .48^a^ | .12^a^ | .104^a^ |  |

^a^ Data are normally distributed. ^b^ Post hoc pairwise comparisons versus baseline *P<*.05. ^c^ Post hoc pairwise comparisons versus baseline *P<*.01. ^d^ Post hoc pairwise comparisons versus baseline *P<*.001. ^e^ Δ values represent the unadjusted improvement within each subgroup. They should not be used for direct statistical comparison between subgroups due to baseline differences.

T1, baseline assessment; T2, 1-week follow-up; T3, 1-month follow-up; T4, 3-month follow-up; C, the Conventional Treatment Group (n=14); D, the Digital Treatment Group (n=13); CMS, the Constant-Murley Score; UCLA, the University of California Los Angeles Shoulder Scale; NRS, Numerical Rating Scale.

Table S5. Results of post hoc pairwise comparisons among subgroups in a randomized controlled trial focusing on rotator cuff injuries at Pudong Gongli Hospital, Shanghai University of Medicine & Health Sciences, Shanghai, China, 2025.

|  | Subgroup(I) | Subgroup(J) | Mean Difference (I-J) | Std. Error | *P* value | 95% CI |
| --- | --- | --- | --- | --- | --- | --- |
| Flexion  (Group D)^a^ | 1 | 2 | 35.63 | 8.097 | .008 | 11.78 to 59.47 |
|  |  | 3 | 16.54 | 4.058 | .004 | 5.71 to 27.37 |
|  | 2 | 1 | -35.62 | 8.097 | .008 | -59.47 to -11.78 |
|  |  | 3 | -19.09 | 9.058 | .135 | -43.7 to 5.52 |
|  | 3 | 1 | -16.54 | 4.058 | .004 | -27.37 to -5.71 |
|  |  | 2 | 19.09 | 9.058 | .135 | -5.52 to 43.7 |
| Abduction  (Group D)^a^ | 1 | 2 | 44.79 | 6.208 | <.001 | 27.57 to 62.01 |
|  |  | 3 | 20.9 | 4.318 | <.001 | 9.94 to 31.86 |
|  | 2 | 1 | -44.79 | 6.208 | <.001 | -62.01 to -27.57 |
|  |  | 3 | -23.89 | 6.788 | .01 | -41.9 to -5.88 |
|  | 3 | 1 | -20.9 | 4.318 | <.001 | -31.86 to -9.94 |
|  |  | 2 | 23.89 | 6.788 | .01 | 5.88 to 41.9 |
| Internal rotation  (Group D)^b^ | 1 | 2 | 32.157 | 7.378 | .001 | 13.277 to 51.037 |
|  |  | 3 | 24.48 | 6.247 | .002 | 8.493 to 40.467 |
|  | 2 | 1 | -32.157 | 7.378 | .001 | -51.037 to -13.277 |
|  |  | 3 | -7.677 | 6.203 | .681 | -23.551 to 8.197 |
|  | 3 | 1 | -24.48 | 6.247 | .002 | -40.467 to -8.493 |
|  |  | 2 | 7.677 | 6.203 | .681 | -8.197 to 23.551 |
| UCLA  (Group D)^a^ | 1 | 2 | 4.94 | 1.674 | .046 | 0.09 to 9.8 |
|  |  | 3 | 1.83 | 0.58 | .014 | 0.35 to 3.31 |
|  | 2 | 1 | -4.94 | 1.674 | .046 | -9.8 to -0.09 |
|  |  | 3 | -3.12 | 1.722 | .225 | -8 to 1.77 |
|  | 3 | 1 | -1.83 | 0.58 | .014 | -3.31 to -0.35 |
|  |  | 2 | 3.12 | 1.722 | .225 | -1.77 to 8 |
| Abduction  (Group C)^a^ | 1 | 2 | 56.49 | 6.795 | <.001 | 38.33 to 74.66 |
|  |  | 3 | 36.57 | 7.704 | <.001 | 16.83 to 56.31 |
|  | 2 | 1 | -56.49 | 6.795 | <.001 | -74.66 to -38.33 |
|  |  | 3 | -19.92 | 9.25 | .103 | -43.25 to 3.41 |
|  | 3 | 1 | -36.57 | 7.704 | <.001 | -56.31 to -16.83 |
|  |  | 2 | 19.92 | 9.25 | .103 | -3.41 to 43.25 |

^a^ The Games-Howell post hoc test was applied because the assumption of homogeneity of variance was violated. ^b^ The Bonferroni post hoc test was applied for multiple comparisons following a significant ANCOVA result, as the assumption of homogeneity of variance was met.

Table S6. Baseline characteristics of patients with NRS=3 versus NRS≥4 in a randomized controlled trial focusing on rotator cuff injuries at Pudong Gongli Hospital, Shanghai University of Medicine & Health Sciences, Shanghai, China, 2025.

| Characteristics | | NRS=3 (n=10) | NRS≥4 (n=50) | *P* value |
| --- | --- | --- | --- | --- |
| Gender | Male, n(%) | 2(20) | 17(34) | .385 |
|  | Female, n(%) | 8(80) | 33(66) |  |
| Injured side | Left, n(%) | 2(20) | 14(28) | .602 |
|  | Right, n(%) | 8(80) | 36(72) |  |
| Age | | 55.5(46.5,69.5) | 62(50,70) | .599 |
| Duration | | 4.5(3,6.25) | 3(3,6) | .393 |
| CMS | | 80(69.75,89.25) | 72(62,80) | .088^a^ |
| UCLA | | 24.5(18.25,26.25) | 20(16,22) | .068^a^ |
| Flexion | | 145(120,151.25) | 130(110,151.25) | .485 |
| Abduction | | 135(97.5,155) | 110(90,150) | .47 |
| External rotation | | 75(40,90) | 70(58.75,90) | .968 |
| Internal rotation | | 40(20,90) | 40(20,50) | .352 |

^a^ Data are normally distributed.

CMS, the Constant-Murley Score; UCLA, the University of California Los Angeles Shoulder Scale.

Table S7. Baseline comparison between the Conventional Treatment Group and the Digital Treatment Group after excluding patients with NRS=3 in a randomized controlled trial focusing on rotator cuff injuries at Pudong Gongli Hospital, Shanghai University of Medicine & Health Sciences, Shanghai, China, 2025.

| Characteristics | Gender | | Injured side | | Age | Duration |
| --- | --- | --- | --- | --- | --- | --- |
|  | Male, n(%) | Female, n(%) | Left, n(%) | Right, n(%) |  |  |
| Group C | 9(40.9) | 13(59.1) | 6(27.3) | 16(72.7) | 63(54.5,70) | 3(3,6) |
| Group D | 8(28.6) | 20(71.4) | 8(28.6) | 20(71.4) | 61.5(47.25,68) | 3(3,6) |
| *P* value | .361 | | .919 | | .318 | .857 |

Group C, the Conventional Treatment Group (n=22); Group D, the Digital Treatment Group (n=28).

Table S8. Sensitivity analysis of primary outcomes after excluding patients with NRS=3 in a randomized controlled trial focusing on rotator cuff injuries at Pudong Gongli Hospital, Shanghai University of Medicine & Health Sciences, Shanghai, China, 2025.

| Groups | | T1 | T2 | T3 | T4 | Δ |
| --- | --- | --- | --- | --- | --- | --- |
| CMS | C | 71.5(65.75,75.5) | 78(70,82)^d^ | 81(73,86)^d^ | 85.5(77.75,90.25)^d^ | 15.41±7.34 |
|  | D | 73(59.5,82.5) | 79(73,87.5)^d^ | 88(82,91.75)^d^ | 91(86.75,96)^d^ | 19.14±9.392 |
|  | *P* value | .328 | .584 | .014 | .003 |  |
| UCLA | C | 21(16.75,22) | 27.5(24.25,30)^d^ | 29(25,30)^d^ | 30(28.25,33)^d^ | 10.36±2.718 |
|  | D | 19(16,22.75) | 27(24.25,31.75)^d^ | 31.5(28,33)^d^ | 33(31.25,35)^d^ | 13.04±3.394 |
|  | *P* value | .855^a^ | .716 | .018 | <.001 |  |
| NRS | C | 5(4,7) | 4(3,5)^d^ | 3(2.75,4.25)^d^ | 2(2,3)^d^ | 2.95±1.463 |
|  | D | 5(4,6) | 4(2.25,4)^d^ | 2(1,2.75)^d^ | 0.5(0,2)^d^ | 4.18±1.307 |
|  | *P* value | .405 | .307 | <.001 | <.001 |  |
| Flexion | C | 125(110,151.25) | 132.5(120,156.25)^b^ | 140(128.75,160)^d^ | 150(130,165)^c^ | 19.09±14.69 |
|  | D | 130(110,172.5) | 140(120,180) | 155(140,180)^d^ | 170(150,180)^d^ | 28.39±23.176 |
|  | *P* value | .637 | .425 | .05 | .012 |  |
| Abduction | C | 105(90,150) | 115(98.75,155)^b^ | 122.5(107.5,155)^b^ | 127.5(115,161.25)^c^ | 20.05±21.034 |
|  | D | 115(92.5,150) | 122.5(102.5,172.5) | 150(116.25,180)^d^ | 152.5(142.5,180)^d^ | 31.43±22.927 |
|  | *P* value | .276 | .371 | .12 | .005 |  |
| External Rotation | C | 67.5(50,90) | 70(60,90)^b^ | 70(60,90)^c^ | 70(70,90)^c^ | 10±15.43 |
|  | D | 70(60,90) | 90(70,90)^c^ | 90(72.5,90)^c^ | 90(90,90)^d^ | 15.89±15.756 |
|  | *P* value | .246 | .019 | .018 | .001 |  |
| Internal Rotation | C | 32.5(11.25,60) | 40(23.75,60)^b^ | 42.5(20,52.5) | 47.5(23.75,60) | 7.5±20.163 |
|  | D | 42.5(20,50) | 47.5(30,60)^b^ | 55(40,70)^c^ | 62.5(45,87.5)^d^ | 21.79±23.5 |
|  | *P* value | .587 | .408 | .029^a^ | .012 |  |

^a^ Data are normally distributed. ^b^ Post hoc pairwise comparisons versus baseline *P<*.05. ^c^ Post hoc pairwise comparisons versus baseline *P<*.01. ^d^ Post hoc pairwise comparisons versus baseline *P<*.001.

T1, baseline assessment; T2, 1-week follow-up; T3, 1-month follow-up; T4, 3-month follow-up; C, the Conventional Treatment Group (n=22); D, the Digital Treatment Group (n=28); CMS, the Constant-Murley Score; UCLA, the University of California Los Angeles Shoulder Scale; NRS, Numerical Rating Scale.
